# Quality control prediction of electrolytic copper using novel hybrid nonlinear analysis algorithm

**DOI:** 10.1038/s41598-023-44546-0

**Published:** 2023-10-16

**Authors:** Yuzhen Su, Weichuan Ye, Kai Yang, Meng Li, Zhaohui He, Qingtai Xiao

**Affiliations:** 1 Department of Quality Management, Inspection and Testing, Yibin University, Yibin, 644000 Sichuan People’s Republic of China; 2 State Key Laboratory of Complex Nonferrous Metal Resources Clean Utilization, Kunming University of Science and Technology, Kunming, 650093 Yunnan People’s Republic of China; 3 Faculty of Metallurgical and Energy Engineering, Kunming University of Science and Technology, Kunming, 650093 People’s Republic of China; 4 Department of Electrical and Computer Engineering, University of Central Florida, Orlando, FL 32816 USA; 5 Design & Research Institute of Kunming University of Science and Technology Co. Ltd., Kunming, 650051 People’s Republic of China; 6 State Environmental Protection Key Laboratory of Mineral Metallurgical Resources Utilization and Pollution Control, Ministry of Ecology and Environment, Wuhan, Hubei 430081 People’s Republic of China

**Keywords:** Characterization and analytical techniques, Chemical engineering

## Abstract

Traditional linear regression and neural network models demonstrate suboptimal fit and lower predictive accuracy while the quality of electrolytic copper is estimated. A more dependable and accurate model is essential for these challenges. Notably, the maximum information coefficient was employed initially to discern the non-linear correlation between the nineteen factors influencing electrolytic copper quality and the five quality control indicators. Additionally, the random forest algorithm elucidated the primary factors governing electrolytic copper quality. A hybrid model, integrating particle swarm optimization with least square support vector machine, was devised to predict electrolytic copper quality based on the nineteen factors. Concurrently, a hybrid model combining random forest and relevance vector machine was developed, focusing on primary control factors. The outcomes indicate that the random forest algorithm identified five principal factors governing electrolytic copper quality, corroborated by the non-linear correlation analysis via the maximum information coefficient. The predictive accuracy of the relevance vector machine model, when accounting for all nineteen factors, was comparable to the particle swarm optimization—least square support vector machine model, and surpassed both the conventional linear regression and neural network models. The predictive error for the random forest-relevance vector machine hybrid model was notably less than the sole relevance vector machine model, with the error index being under 5%. The intricate non-linear variation pattern of electrolytic copper quality, influenced by numerous factors, was unveiled. The advanced random forest-relevance vector machine hybrid model circumvents the deficiencies seen in conventional models. The findings furnish valuable insights for electrolytic copper quality management.

## Introduction

Although pyro refining could be used to yield copper products with minimal impurities, these products potentially did not meet the stringent high-quality standards for copper. Consequently, most of the crude copper often undergo electrorefining to eliminate the impurities that are resistant to the pyrometallurgical refinement, thereby enhancing the quality of electrolytic copper^1^. Typically, copper from the anode dissolves into the solution under direct current. Preferentially, copper from this solution precipitates on the cathode, resulting in what is termed as electrolytic copper. During this electrolysis procedure of copper, operating parameters have traditionally been determined based on the experience of operators, introducing significant subjectivity and arbitrariness, and being susceptible to various disturbances. However, the production process could yield inconsistent quality of electrolytic copper, evidenced by a low proportion of first-grade products which were characterized by the uneven copper distribution, frequent fins, and granular protrusions^2^. Hence, it would be imperative to investigate and control the factors influencing the quality of electrolytic copper to attain the improved outcomes. Current research trend is merging quality prediction with control and it is transitioning from conventional, reactive methodologies to proactive quality prediction techniques. These proactive approaches allow for early detection of potential production issues, facilitating the timely remediation and minimizing the quality degradation. Therefore, predictive control over electrolytic copper quality would stand as a pivotal concern in producing cathode copper with high-purity.

There are growing appeals for predictive control over electrolytic copper quality. For instance, Zhao et al.^3^ utilized atomic force microscopy and image scaling analysis technique to predict the influence of current density, temperature, and leveling agent on the morphology of electrolytically produced copper. Notwithstanding, the main issue is that the production process of electrolytic copper often encompasses numerous intricate physical and chemical reactions. The interplay within these reactions bestows the copper electrolysis process with heightened nonlinearity and complexity, rendering traditional statistical methods for quality prediction and control challenging. As a result, limited literature addresses the quality issues of copper electrolysis. Moreover, it is noteworthy that publications on quality prediction have been increasingly prevalent in recent years, with an ascending trend in publication counts: 9736 (2018), 10,945 (2019), 12,312 (2020), 12,182 (2021), and 12,334 (2022)^4–7^. Research methodologies on product quality has been broadly categorized into (1) conventional statistical process control theories which are exemplified by classic control charts and (2) contemporary intelligent prediction and control algorithms, which are notably epitomized by artificial neural networks (ANN). Control charts exhibit efficacy in large-scale production due to vast data mean ranges, significant offsets, and operational simplicity^8,9^. However, the sensitivity of control charts would wane with the diminutive average or offset of production data. Intelligent algorithms were rooted in principles or mechanisms of natural phenomena or entities and then predominantly employed for earlier study on the product predictions^10^. Conversely, the early research initially leaned towards the traditional linear regression and conventional neural network models^11^. Currently, the artificial neural network methodologies garner substantial interest globally, spurring the evolution of diverse research trajectories. For instance, the widely used intelligent algorithms consist of support vector machine (SVM)^12^, particle swarm optimization (PSO)^13^, random forest (RF)^14^, relevance vector machine (RVM)^15^, and other machine learning algorithms^16–19^. Such advancements facilitate the effective integration of intelligent algorithms within the engineering domain (i.e., chemical engineering or metallurgical engineering), resulting in innovative avenues for industrial research. In addition, this not only augments prediction accuracy but also broadens applicability.

Numerous studies focus on the modeling and design of industrial process^20,21^. For instance, Zang et al.^22^ developed an Arrhenius model coupled with a radial basis function (RBF) neural network to forecast oxidative alterations in whole egg powder. In a distinct approach, Ma et al.^23^ integrated partial least squares regression analysis of water quality with morphological spatial pattern analysis data to holistically assess the effects of land-use variations and landscape patterns on basin water quality. Similarly, Wang et al.^24^ employed spatially adaptive machine learning models to predict water quality in Hong Kong. Artificial neural networks present a promising avenue for delving deeper into industrial processes while traditional methodologies have not yielded commendable outcomes^25–28^. Collectively, these investigations underscore the efficacy of neural networks for industrial parameter predictions. However, neural networks also exhibit inherent limitations including the prerequisite for predefined network structures, susceptibility to local optima, and suboptimal generalization capabilities^29^. Predominant quality prediction techniques often emphasize model-centric approaches, inadvertently sidelining direct influencers including production equipment, operational environment, and workforce dynamics. For addressing this issue, Zhang et al.^30^ amalgamated the principal component analysis (PCA) technique with the support vector machine model, devising a quality prediction framework tailored for diverse, small-batch products. Conversely, Bai et al.^31^ harnessed principal component analysis to distill low-dimensional data, subsequently implementing support vector machine for modeling desensitization data from China’s Tianchi Big Data Contest. Components that are not relevant to parameter estimation can be rejected by PCA^32^. However, the PCA-derived principal components might not yield optimal results while non-Gaussian distributions was confronted. As mentioned, moreover, the intricate physical and chemical interplay within the electrolytic process of copper often manifests profound nonlinear traits. Augmenting this paradigm, He et al.^33^ introduced a product quality model grounded in relevance vector machine, transforming raw input into feature-rich space via kernel functions, offering a promising framework for quality prediction and control of electrolytic copper.

As highlighted, it remains challenging to effectively integrate diverse and disjointed factors into the quality prediction model of electrolytic copper even when considering the significance of various influencing factors to enhance the predictive accuracy for electrolytic copper quality. Notably, there exists a paucity of impactful research insights on utilizing known quantitative factors in the electrolytic copper production process to mitigate data wastage and augment prediction precision. This work aims to prioritize high-quality, energy-efficient production process of electrolytic copper by conducting multi-factorial, small-batch industrial experiments. Specifically, a thorough literature analysis on copper quality prediction and control is undertaken by employing both traditional and contemporary methodologies, culminating in the formulation and establishment of a novel predictive model for copper quality. The innovation of this work resides in the pioneering identification of five primary control factors impacting electrolytic copper quality using the random forest algorithm. Moreover, a hybrid model integrating particle swarm optimization with least square support vector machine (PSO-LSSVM) is introduced for predicting electrolytic copper quality based on the nineteen associated factors. Concurrently, a hybrid model combining random forest with relevance vector machine (RF-RVM) is crafted for quality prediction using these primary control factors. Then, the interference of extraneous variables on electrolytic copper quality is minimized by discerning the effect of these main control factors as realistic as possible, laying foundational insights into the mechanisms influencing electrolytic copper quality. The capabilities of inherent inadequacies and suboptimal prediction of conventional linear regression and neural network models are addressed. The newly introduced hybrid models bolster the dependability of predictions pertaining to electrolytic copper quality. Hence, the innovative strategy for in-depth exploration of industrial site data bears significant implications for the precise control of electrolytic copper quality when the specific attributes of electrolytic copper control objects and the exigencies of production are considered.

The structure of this article unfolds as follows: Section "Method and data" elucidates methods and data, encompassing the least square support vector machine, relevance vector machine, evaluation indices, and data description. Section "Result and discussion" delves into the primary controlling factors and associated prediction models. Conclusions are drawn in section “Conclusion”.

## Method and data

### LSSVM

Least squares support vector machine is a modified version of the conventional support vector machine^34^. It offers the benefits of straightforward computation, effortless operation, rapid learning, and convenient implementation. In terms of implementation, the linear regression function $y(x)$ of least squares support vector machine is defined by^35^

1 $$y\left(x\right)=w\cdot \varphi \left(x\right)+b,$$

where $w$ represents the weight vector, $\varphi (x)$ stands for the mapping function, and $b$ signifies the offset vector. By leveraging the structural risk minimization principle, the optimization challenge of LSSVM is articulated as^35^

2 $$\left\{\begin{array}{l}\underset{w, b,e}{\mathrm{min}}J(w,e)=\frac{1}{2}{w}^{\mathrm{T}}w+\frac{1}{2}\gamma \sum_{k=1}^{N}{e}_{k}^{2}\\ {y}_{k}={w}^{\text{T}}\varphi \left({x}_{k}\right)+b+{e}_{k}\end{array},\right.$$

where $k$ ranges from 1 to $N$, $\gamma$ refers to the penalty coefficient, ${e}_{k}$ refers to the error in fitness, and $b$ refers to the threshold value. To address this issue, the Lagrange function is formulated, introducing the Lagrange multiplier $\alpha$ such that $\alpha \ge 0$. Then,

3 $$L(w,b,e,\alpha )=J(w,e)-\sum_{k=1}^{N}{\alpha }_{k}\left[{w}^{\mathrm{T}}\varphi ({x}_{k})+b+{e}_{k}-{y}_{k}\right],$$

Taking partial derivatives of the above yields and then^35^

4 $$\left\{\begin{array}{l}\begin{array}{l}\begin{array}{ccc}\frac{\partial L}{\partial w}=0& \Rightarrow & w=\sum_{k=1}^{N}{\alpha }_{k}\varphi ({x}_{k})\end{array}\\ \begin{array}{ccc}\frac{\partial L}{\partial b}=0& \Rightarrow & \sum_{k=1}^{N}{\alpha }_{k}=0\end{array}\\ \begin{array}{ccc}\frac{\partial L}{\partial {e}_{k}}=0& \Rightarrow & {\alpha }_{k}=\gamma {e}_{k}\end{array}\\ \begin{array}{ccc}\frac{\partial L}{\partial \alpha }=0& \Rightarrow & {w}^{\mathrm{T}}\end{array}\varphi ({x}_{k})+b+{e}_{k}-{y}_{k}=0\end{array}\end{array},\right.$$

where $k$ ranges from 1 to $N$. Then, $w$ and ${e}_{k}$ are excluded. A kernel function is introduced by

5 $$K\left({x}_{m},{x}_{n}\right)={\varphi \left({x}_{m}\right)}^{\mathrm{T}}\varphi \left({x}_{n}\right),$$

where both $m$ and $n$ range from 1 to $N$. This leads to the following matrix equation which is given by

6 $$\left[\begin{array}{cc}0& {1}^{\mathrm{T}}\\ 1& \Omega +{\gamma }^{-1}I\end{array}\right]\left[\begin{array}{c}b\\ \boldsymbol{\alpha }\end{array}\right]=\left[\begin{array}{c}0\\ y\end{array}\right],$$

where ${1}^{\mathrm{T}}=[\mathrm{1,1},\cdots ,1]$ and $\boldsymbol{\alpha }={[{\alpha }_{1},{\alpha }_{2},\cdots ,{\alpha }_{N}]}^{\text{T}}$. In this work, the radial basis function was chosen as the kernel function, which is given by

7 $$K\left(x,{x}_{k}\right)=\mathrm{exp}\left[-\frac{{\left(x-{x}_{k}\right)}^{2}}{2{\sigma }^{2}}\right],$$

where $\sigma$ refers to the width of kernel function. The LSSVM predictive model is subsequently derived by

8 $$y\left(x\right)=\sum_{k=1}^{N}{\alpha }_{k}K\left(x,{x}_{k}\right)+b.$$

Hence, it becomes evident that the judicious selection of parameters in the LSSVM optimization model profoundly influences the intricacy and precision of model. Consequently, both the penalty coefficient $\gamma$ and the kernel coefficient $\sigma$ hold significant importance.

### RVM

Relevance vector machine is a relatively new approach that has not been used widely in metallurgical process. Both relevance vector machine and support vector machine could utilize the kernel functions to convert the challenge of linear inseparability in lower-dimensional space to that of linear partitioning in higher-dimensional space^36,37^. The salient distinction between relevance vector machine and support vector machine lies in that relevance vector machine inherits the similar decision function and the choice of kernel function is more flexible. Hence, the classification function could attain its peak on the likelihood function value of the training set. For classification of relevance vector machine, the Laplace method could be employed for impending approximation. Both the weight posterior probability $p(w|t,\alpha )$ and the marginal likelihood function $p(t|\alpha )$ could be derived through integration. Consequently, the classification issue of relevance vector machine could be reframed as a regression issue.

### Evaluation indices

Here, the prediction results are assessed using mean absolute error (MAE) and root mean square error (RMSE). In fact, mean absolute error offers an accurate representation of prediction value discrepancies, while root mean square error quantifies the deviation between forecasted values and actual ones^38^. The computation for the $j$-th component of the electrolytic copper mass is given by^38^

9 $$\mathrm{MAE}\left(j\right)=\frac{1}{N}\sum_{k=1}^{N}\left|{y}_{j}\left(k\right)-{\widehat{y}}_{j}\left(k\right)\right|,$$

and

10 $$\mathrm{RMSE}\left(j\right)=\sqrt{\frac{1}{N}\sum_{k=1}^{N}{\left({y}_{j}\left(k\right)-{\widehat{y}}_{j}\left(k\right)\right)}^{2}},$$

where ${y}_{j}(k)$ denotes the actual value of the $j$-th component of the electrolytic copper mass for the $k$-th experimental instance, and ${\widehat{y}}_{j}(k)$ signifies the predicted value for the same component in the $k$-th experimental instance.

### Data description

The used experimental data were sourced from publicly available literature. Nineteen primary factors influencing product quality were identified from the product data, with each factor comprising $N=36$ representative test data points. An investigation based on technical standards was conducted to examine the various factors influencing the quality of electrolytic copper. The primary quality indices for electrolytic copper include anode copper periphery (X_1_), anode copper surface (X_2_), starting piece periphery (X_3_), starting piece surface (X_4_), starting piece toughness (X_5_), Cu content in anode copper chemical composition (X_6_), As content in anode copper chemical composition (X_7_), cell voltage (X_8_), current density (X_9_), electro-hydraulic temperature (X_10_), electro-hydraulic flow (X_11_), number of short circuits (X_12_), Cu content in electro-hydraulic composition (X_13_), H_2_SO_4_ content in electro-hydraulic composition (X_14_), As in electro-hydraulic composition (X_15_), gelatin content in additives (X_16_), thiourea content in additives (X_17_), casein content in additives (X_18_), and hydrochloric acid content in additives (X_19_). The quality of electrolytic copper, derived from both its chemical composition and physical specification indices, was deconstructed to focus specifically on its components Cu and As. The resulting quality components were defined as electrolytic copper periphery (Y_1_), electrolytic copper surface (Y_2_), electrolytic copper toughness (Y_3_), copper content in electrolytic copper (Y_4_), and arsenic content in electrolytic copper (Y_5_).

**Figure 1:**
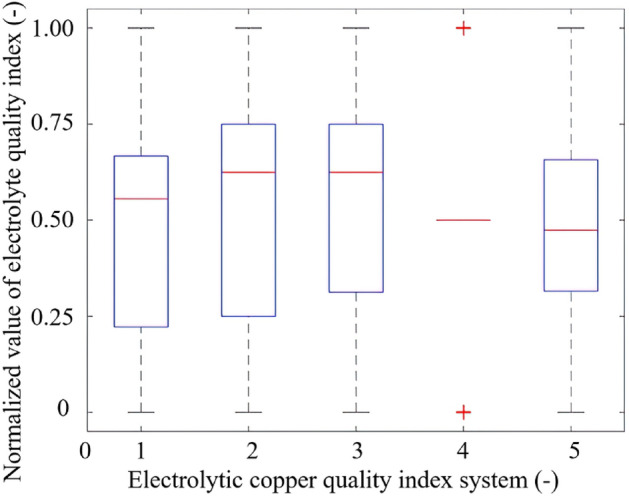
Box plot of the normalized value of five-status indicator system of electrolytic copper quality.

**Figure 2:**
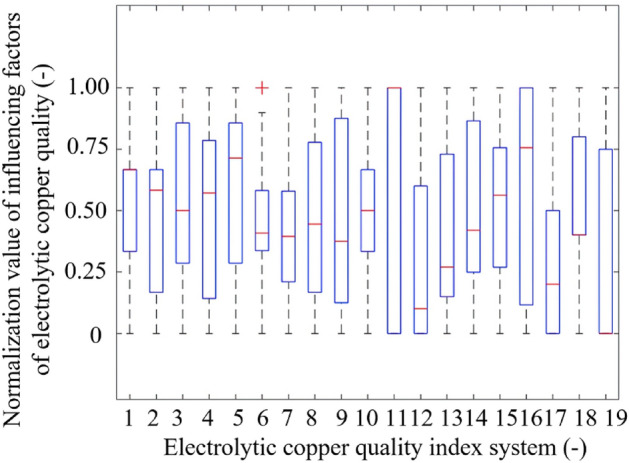
Box plot of the normalized values of nineteen factors affecting the electrolytic copper quality.

In order to enhance the convergence speed and accuracy of the proposed models, data normalization was first executed. Given that all data points are fixed, the min–max procedure refers to the linearly transforms for the original data, ensuring that results fall within the interval $[0, 1]$. Consequently, min–max standardization was employed for data processing, represented by the subsequent equations. For $i=1, 2,\cdots , 19 \mathrm { ~and~ } j=1, 2, 3, 4, 5$, the transition variable are given by

11 $$x_{i} = \frac{{X_{i} - X_{i}^{{{\text{min}}}} }}{{X_{i}^{{{\text{max}}}} - X_{i}^{{{\text{min}}}} }},$$

and

12 $$y_{j} = \frac{{Y_{j} - Y_{j}^{{{\text{min}}}} }}{{Y_{j}^{{{\text{max}}}} - Y_{j}^{{{\text{min}}}} }},$$

where ${x}_{i}$ denotes the transformed factors affecting electrolytic copper quality, and ${y}_{j}$ represents the transformed quality of electrolytic copper. Additionally, ${X}_{i}$ refers to the factors impacting electrolytic copper quality prior to the transformation, and ${Y}_{j}$ indicates the quality of electrolytic copper before said transformation. ${X}_{i}^{\text{max}}$ and ${X}_{i}^{\text{min}}$ refer to the maximum and minimum, respectively, among thirty-six test data sets for the $i$-th quality-affecting factor of electrolytic copper. Similarly, ${Y}_{j}^{\text{max}}$ and ${Y}_{j}^{\text{min}}$ refer to the maximum and minimum, respectively, among thirty-six test data sets for the $j$-th mass component of electrolytic copper. Normalized box plots of the quality of electrolytic copper and its influencing factors are depicted in Figs. 1 and 2. The graphics reveal outliers for both Y_4_ and X_6_, with data values exceeding the upper and lower boundaries. The experimental data pertaining to electrolytic copper quality and its associated factors present varying medians. With the exceptions of X_1_, X_11_, X_18_, and X_19_, the data distribution is relatively uniform. Thus, from a macroscopic data perspective, the relationship between the influencing factors and the quality of electrolytic copper appears intricately complex and profoundly nonlinear.

## Result and discussion

### Non-linear correlation analysis

Based on relevant research findings, the quality of electrolytic copper is influenced by various factors including personnel, equipment, environment, operation, and raw materials. These elements exhibit a nonlinear relationship, mutually interacting and constraining one another, collectively determining the quality of electrolytic copper. The traditional linear statistical approach faces challenges in deciphering these multifaceted influencing factors. Notably, researchers from the Broad Institute at Harvard University introduced a robust statistical method rooted in the maximal information coefficient (MIC), highlighting significant relationships^39^. The values of MIC ranging from 0.90 to 1.00 signify an exceptionally high correlation, the values between 0.70 and 0.90 denote a high correlation, the values between 0.40 and 0.70 suggest a moderate correlation, the values between 0.20 and 0.40 represent a low correlation; values from 0.10 to 0.20 indicate a very low correlation, and the values less than 0.10 imply a lack of correlation. Consequently, this work employs MIC to quantify the nonlinear association among factors influencing the quality of electrolytic copper, offering valuable insights into the critical determinants for quality management of electrolytic copper.

**Figure 3:**
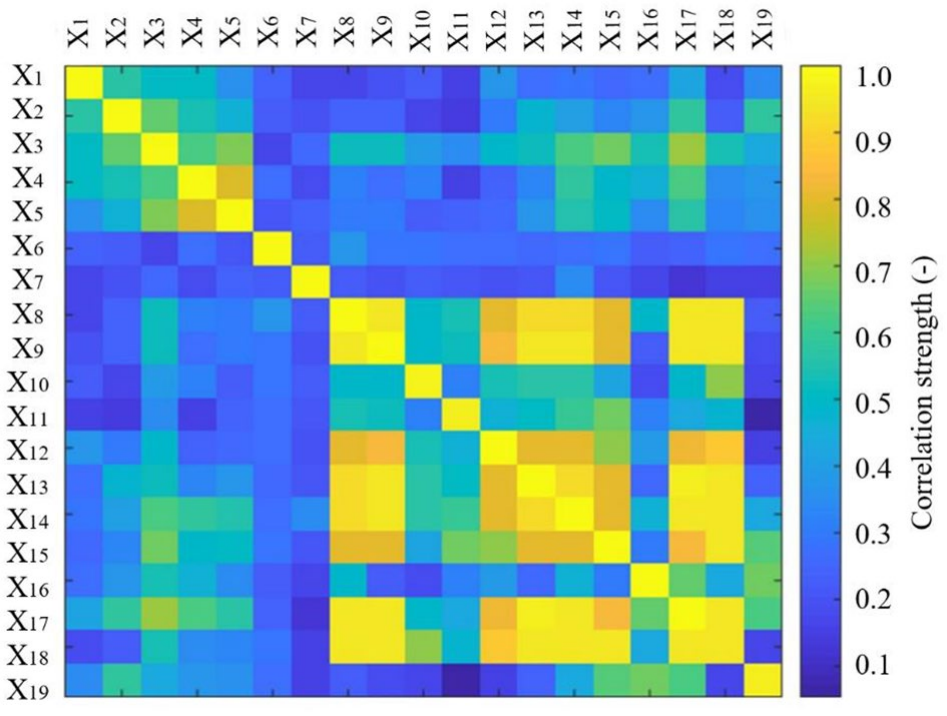
Non-linear analysis results of factors affecting the electrolytic copper quality using maximal information coefficient.

Calculations were conducted using MIC method via the popular mathematical software program based on thirty-six sets of experimental data encompassing nineteen distinct influencing factors. The resultant data are depicted in Fig. 3. From this figure, it can be observed that the yellow area at the bottom right occupies a larger area. The yellow color indicates a strong correlation between the two factors. Specifically, the computed MIC values between X_8_ and X_9_, X_13_, X_14_, X_17_, X_18_ are 0.94, 0.92, 0.92, 0.94, 0.94 respectively. Similarly, the MIC values for X_9_, and X_13_, X_4_, X_17_, X_18_ are 0.94, 0.94, 0.94, 0.94, and so on. Factors X_17_ and X_18_ exhibit a MIC value of 0.94, indicating a notably high correlation (i.e., MIC values exceeding 0.90). In contrast, the MIC value between X_3_ and X_17_ stands at 0.71, while for X_4_ and X_5_ it is 0.79. Additionally, the values for X_8_ and X_12_, X_15_ are 0.80 and 0.81 respectively. These relationships reflect a high correlation, as MIC values range from 0.70 to below 0.90. These results emphasize that harnessing the variability characteristics of electrolytic copper data can enhance the analysis of the correlation among its quality-affecting factors. It is also found that the maximum information coefficient is apt for exploring correlations amid complex variables, exemplified by fluctuations in factors influencing electrolytic copper quality.

In fact, the change of one influencing factor of electrolytic copper often leads to the change of other influencing factors of electrolytic copper in terms of the accrual production process of hydrometallurgy. Furthermore, these changes are difficult to observe during the copper electrolysis procedure. To solve this problem of electrolytic copper through the industrial testing method is not only costly but also difficult to achieve the expected object. Nevertheless, the relationship between various factors is intuitively displayed through the calculation of maximal information coefficient. Hence, the dynamic correlations among diverse influencing factors are holistically evaluated in formulating quality control protocols for electrolytic copper, minimizing the undue focus on isolated variables.

### Primary influencing factors

Random forest is one of the most influential techniques in machine learning^40^. This method utilizes multiple decision trees to facilitate comprehensive classification, correlation analysis, prediction, and data interpretation^41^. In this work, the dependent variable pertains to the quality of electrolytic copper, representing the target for decision classification. Conversely, the independent variables encompass a range of factors potentially impacting the quality of electrolytic copper, such as the starting sheet quality and the chemical composition of the anode copper. These variables serve as predictors for the dependent variable. Constructing the random forest model involves the following several steps. (1) Extracting training samples from the original dataset using the Bootstrap method, subsequently establishing $n$ trees. (2) During the tree generation procedure, variables with number of $m$ are randomly chosen at each tree node, from which those exhibiting the highest classification efficacy are selected for data classification. (3) The data excluded during the Bootstrap extraction serves as the test sample to appraise the performance of each tree. Together, the trees with number of $n$ constitute a random forest for data prediction.

The random forest algorithm was employed to evaluate the significance of factors impacting the variability in quality of electrolytic copper. The results are delineated in Table 1. For the electric copper periphery (Y_1_), according to this table, the primary influential factors include the periphery of the starting sheet (X_3_), the additive with thiourea (X_17_), the electro-hydraulic component with H_2_SO_4_ (X_14_), the electro-hydraulic component with Cu (X_13_), and the additive with casein (X_18_). Regarding the copper surface (Y_2_), the principal determinants are additives with thiourea (X_17_), the periphery of the starting sheet (X_3_), additives with casein (X_18_), cell voltage (X_8_), and electro-hydraulic components with Cu (X_13_). For the toughness of electrolytic copper (Y_3_), significant factors encompass the periphery of the starting sheet (X_3_), the additive with thiourea (X_17_), the electro-hydraulic component with H_2_SO_4_ (X_14_), cell voltage (X_8_), and the additive with casein (X_18_). In terms of electro copper content (Y_4_), the prevailing factors are the electro-hydraulic component with Cu (X_13_), the electro-hydraulic component with H_2_SO_4_ (X_14_), the number of short circuits (X_12_), the additive with thiourea (X_17_), and the additive with gelatin (X_16_). For arsenic in electro copper (Y_5_), the primary influencers are H_2_SO_4_ content (X_14_), Cu content (X_13_), current density (X_9_), casein content (X_18_), and cell voltage (X_8_). Subsequent investigations corroborated the nonlinear correlations deduced by maximal information coefficient, aligning with the primary determinants of electrolytic copper quality as identified by the random forest approach.

**Table 1:** Importance of factors affecting the electrolytic copper quality by using random forest algorithm.

Factor	Y_1_	Y_2_	Y_3_	Y_4_	Y_5_
X_1_	0.1768	0.0443	0.0982	0.0109	0.0289
X_2_	0.1082	0.2148	0.1090	− 0.0845	− 0.1615
X_3_	**0.4294**	**0.4216**	**0.5482**	− 0.0668	0.0514
X_4_	0.2706	0.2784	0.3083	− 0.0911	0.0236
X_5_	0.0070	− 0.0043	0.2108	− 0.0037	0.0323
X_6_	− 0.0576	0.1629	− 0.1159	0.0123	− 0.2284
X_7_	− 0.0176	0.0221	0.0455	− 0.1493	− 0.0904
X_8_	0.3341	**0.3332**	**0.4494**	− 0.0462	**0.2905**
X_9_	0.3143	0.2978	0.3126	0.0578	**0.3633**
X_10_	− 0.0918	0.0752	− 0.0658	− 0.0784	0.1377
X_11_	− 0.0173	0.1276	0.1313	0.0730	0.0569
X_12_	0.1741	0.3005	0.2269	**0.1718**	0.1638
X_13_	**0.3782**	**0.3105**	0.2372	**0.3335**	**0.4300**
X_14_	**0.4089**	0.2490	**0.4499**	**0.2132**	**0.4698**
X_15_	0.1645	0.2480	0.1574	− 0.1110	0.2077
X_16_	0.1814	0.0692	0.1183	**0.0884**	− 0.2003
X_17_	**0.4257**	**0.5400**	**0.4975**	**0.0966**	0.1663
X_18_	**0.3525**	**0.3558**	**0.3412**	0.0280	**0.3438**
X_19_	0.0761	0.2318	0.2354	− 0.0776	0.0281

Significant values are in [boldunderline].

Hence, the primary factors of five quality control indicators for electrolytic copper quality were obtained. Although the quality control of copper electrolysis could be achieved by studying nineteen influencing factors, the acquisition of controlling factors could greatly simplify the research process. Especially in complex industrial production processes, controlling the five primary factors could not only improve production efficiency but also quickly improve product quality probably. At the same time, the acquisition of primary factors also provides fundamental for the prediction of copper electrolytic quality.

### Comparison of prediction methods

The literature details quality indices of electrolytic copper, and nineteen factors influencing this quality were compiled into a sample library. For training and testing, ${N}_{1}$=27 groups of electrolytic copper experimental data constituted the training set, while the remaining ${N}_{2}$=9 groups formed the testing set. For comparative study, various algorithms, namely back propagation neural network, least squares support vector machine, relevance vector machine, and support vector machine enhanced by particle swarm optimization, were employed to develop the control predictive model of electrolytic copper quality. Specifically, the back propagation neural network utilized a three-layer network structure with parameters such as a maximum iteration of 1000, a learning rate of 0.01, a training error threshold of 0.0001, a momentum factor of 0.01, a minimum performance gradient of 10^–6^, and a maximum failure count of 6. For least square support vector machine, the primary computational parameters comprised a kernel width of sig2 = 500, a regularization parameter of gam = 5, with the RBF kernel function selected. Utilizing these methodologies, data from thirty-six actual production instances in the publicly available dataset were modeled and predicted. Predictive outcomes are presented in Table 2. According to this table, notably, parameters for the particle swarm optimization algorithm were pre-established, achieving anticipated optimization outcomes. Furthermore, results derived from the multiple linear regression model in this table are based on linear regression equations pertaining to various attributes of electrolytic copper as sourced from public literature. All other results emanate from the four artificial intelligence algorithms introduced in this work, dedicated to predicting control of electrolytic copper quality.

**Table 2:** Prediction results of electrolytic copper quality by different prediction methods.

Error index	Models				
	MLR	BP	LSSVM	RVM	PSO-LSSVM
MAE	7.8467	0.1847	0.1749	0.0663	0.0634
RMSE	16.8610	0.2854	0.2061	0.1161	0.1017

Observational data indicate that the predictive accuracy of the PSO-LSSVM model significantly surpasses other conventional artificial intelligence techniques, whether it is evaluated using mean absolute error or root mean square error. Nevertheless, the accuracy of relevance vector machine closely trails that of PSO-LSSVM, exhibiting discrepancies of 4.45% and 14.16%, respectively. Such outcomes suggest that the PSO-LSSVM prediction method is suitable for multi-variety and small-batch production forecasting, thus expanding the applicability spectrum of PSO-LSSVM within this domain of hydrometallurgical process.

### Effect of steps on predicting accuracy

The quantity of data, denoted as ${N}_{2}$, utilized for forecasting the quality control status of electrolytic copper remains indeterminate. A sensitivity analysis concerning this data volume is imperative, anchored by the evaluation metrics of mean absolute error and root mean square error. Table 3 shows the influence of prediction step size on prediction of electrolytic copper quality using relevance vector machine. Table 4 shows the influence of prediction step size on prediction of electrolytic copper quality using PSO-LSSVM. As delineated in Tables 3 and 4, for training set proportions of 80%, 85%, 90%, and 95%, the corresponding sample sizes are 29, 31, 32, and 34, while for the testing set, they are 7, 5, 4, and 2, respectively. In this work, the sensitivity associated with the predicted values for electrolytic copper quality control status were investigated. Notably, as the proportion of training set samples to the complete dataset transitions from 80 to 95%, the mean absolute error and root mean square error for the relevance vector machine model of electrolytic copper quality exhibit a pattern of initial decline followed by an ascent, reaching their nadir at 90%.

**Table 3:** Influence of prediction step size on prediction performance of relevance vector machine for electrolytic copper quality.

Error index	The proportion of training sets			
	80%	85%	90%	95%
MAE	0.0801	0.0541	0.0362	0.0575
RMSE	0.1303	0.0954	0.0658	0.0892

**Table 4:** Influence of prediction step size on prediction performance of PSO-LSSVM for electrolytic copper quality.

Error index	The proportion of training sets			
	80%	85%	90%	95%
MAE	0.0772	0.0586	0.0500	0.0611
RMSE	0.1149	0.0833	0.0670	0.0783

Additionally, as the proportion of training set samples relative to the entire dataset shifts from 80 to 95%, the mean absolute error and root mean square error for the PSO-LSSVM model concerning electrolytic copper quality consistently exhibit an initial decline, followed by an increase, with the minimum values observed at 90%. In general, relevance vector machine and the PSO-RVM hybrid model are close to each other in accuracy for predicting copper electrolytic quality. The hybrid PSO-RVM model is slightly more stable than relevance vector machine in the prediction process. The proposed hybrid PSO-RVM model may be a good choice for the production process which needs to consider all the influencing factors. However, the number of factors that are input into a predictive model is not always better. However, the accuracy of the model does not increase with the number of input factors. The objective of industrial processes is to minimize the number of factors used for predicting the desired outcome.

### Prediction of electrolytic copper quality

Based on the presented research findings, five primary control factors were identified among the determinants influencing electrolytic copper quality. Utilizing ${N}_{1}$=32 groups of electrolytic copper experimental data as training sets and the remaining ${N}_{2}$=4 groups as test sets, the RF-RVM model was developed to provide intelligent predictions for metallurgical engineering. Table 5 shows the indictors prediction of electrolytic copper quality using relevance vector machine and RF-RVM models. It can be seen from this table that the prediction accuracy of the two models is satisfactory. For instance, the maximal value of mean absolute error is 0.1352 when the relevance vector machine and the data of electrolytic copper periphery (Y_1_) were used. Conversely, the maximal value of root mean square error is 0.1889 when the relevance vector machine and the data of copper content in electrolytic copper (Y_4_) were used. However, it becomes evident that the relevance vector machine yields a higher error while evaluating the prediction outcomes using the two metrics (i.e., mean absolute error and root mean square error). The hybrid RF-RVM model demonstrates superior predictive performance compared to relevance vector machine, achieving a minimal value of 0.0427 in terms of the error index (i.e., root mean square error) and electrolytic copper periphery (Y_1_), less than 5%. Furthermore, the maximal value of prediction performance between relevance vector machine and RF-RVM model are 0.0925 and 0.0842 both for electrolytic copper periphery (Y_1_). The necessity and merit of the proposed hybrid model is clearly demonstrated by the above.

**Table 5:** Indictors prediction of electrolytic copper quality using relevance vector machine and RF-RVM models.

Error index	MAE		RMSE	
Models	RVM	RF-RVM	RVM	RF-RVM
Y_1_	**0.1352**	0.0427	0.1373	0.0531
Y_2_	0.0883	0.0862	0.0982	0.0956
Y_3_	0.0833	0.0721	0.0948	0.0867
Y_4_	0.1279	0.1048	**0.1889**	0.1822
Y_5_	0.1165	0.0919	0.1277	0.1052

Significant values are in [boldunderline].

Consequently, this novel hybrid model leverages the strengths of the random forest algorithm in extracting features (i.e., five pivotal controlling factors are extracted from nineteen determining factors) of electrolytic copper quality. Specifically, it is used to filter out redundant information among the numerous influencing factors, and the issues of the complexities associated with small samples, high dimensionality, and nonlinearity in hydrometallurgy engineering are adeptly addressed. In addition, the proposed RF-RVM hybrid model not only extracts the primary factors for copper electrolytic process, but also caters to the intelligent or digital development needs of metallurgical enterprises or hydrometallurgy process. In other words, the technologies of digital metallurgical engineering are useful to advance the knowledge on data science, machine learning and computational sciences to tackle metallurgical engineering problems.

## Conclusions

From an in-depth analysis of the production mechanism of copper electrolysis and the consideration of factors such as electrolyte composition and power consumption, a predictive model was established for the quality control of electrolytic copper. The primary findings are as follows. (1) The random forest algorithm effectively delineates the intricate nonlinear relationship between factors that determine electrolytic copper quality. Five pivotal controlling factors of electrolytic copper have been elucidated, further corroborated by nonlinear correlation analysis employing maximal information coefficient. (2) Given an input of all nineteen determining factors, the predictive accuracy of relevance vector machine closely parallels that of the PSO-LSSVM model, with deviations of 4.45% and 14.16% respectively. Notably, it surpasses the conventional multiple linear regression and traditional neural network models in this regard. (3) The introduction of an electrolytic copper quality prediction model, based on the RF-RVM model, yields a prediction error for the test data set that is notably smaller than the relevance vector machine, with the minimum error index registering below 5%. To sum up, in this work, the employed machine learning technique adeptly discerns the latent correlations within the electrolytic copper experimental data, diminishes computational complexity, and demonstrates potential applicability to other quality prediction challenges in various metallurgical processes.

## Data Availability

The datasets utilized and analyzed in the present study are available upon reasonable request from the corresponding author.
